# Parallel detection of chemical reactions in a microfluidic platform using hyperpolarized nuclear magnetic resonance†

**DOI:** 10.1039/d3lc00474k

**Published:** 2023-10-19

**Authors:** Jose Yeste, Marc Azagra, Maria A. Ortega, Alejandro Portela, Gergő Matajsz, Alba Herrero-Gómez, Yaewon Kim, Renuka Sriram, John Kurhanewicz, Daniel B. Vigneron, Irene Marco-Rius

**Affiliations:** a Institute for Bioengineering of Catalonia, The Barcelona Institute of Science and Technology Barcelona Spain imarco@ibecbarcelona.eu; b Department of Radiology and Biomedical Imaging, University of California San Francisco San Francisco California USA; c Graduate program in Bioengineering, University of California, Berkeley and University of California San Francisco California USA

## Abstract

The sensitivity of NMR may be enhanced by more than four orders of magnitude *via* dissolution dynamic nuclear polarization (dDNP), potentially allowing real-time, *in situ* analysis of chemical reactions. However, there has been no widespread use of the technique for this application and the major limitation has been the low experimental throughput caused by the time-consuming polarization build-up process at cryogenic temperatures and fast decay of the hyper-intense signal post dissolution. To overcome this limitation, we have developed a microfluidic device compatible with dDNP-MR spectroscopic imaging methods for detection of reactants and products in chemical reactions in which up to 8 reactions can be measured simultaneously using a single dDNP sample. Multiple MR spectroscopic data sets can be generated under the same exact conditions of hyperpolarized solute polarization, concentration, pH, and temperature. A proof-of-concept for the technology is demonstrated by identifying the reactants in the decarboxylation of pyruvate *via* hydrogen peroxide (*e.g.* 2-hydroperoxy-2-hydroxypropanoate, peroxymonocarbonate and CO_2_). dDNP-MR allows tracing of fast chemical reactions that would be barely detectable at thermal equilibrium by MR. We envisage that dDNP-MR spectroscopic imaging combined with microfluidics will provide a new high-throughput method for dDNP enhanced MR analysis of multiple components in chemical reactions and for non-destructive *in situ* metabolic analysis of hyperpolarized substrates in biological samples for laboratory and preclinical research.

## Abstract 2

We developed a microfluidic device compatible with hyperpolarization-enhanced MR spectroscopic imaging. It measures up to 8 chemical reactions simultaneously with a single hyperpolarized sample.

## Graphical abstract

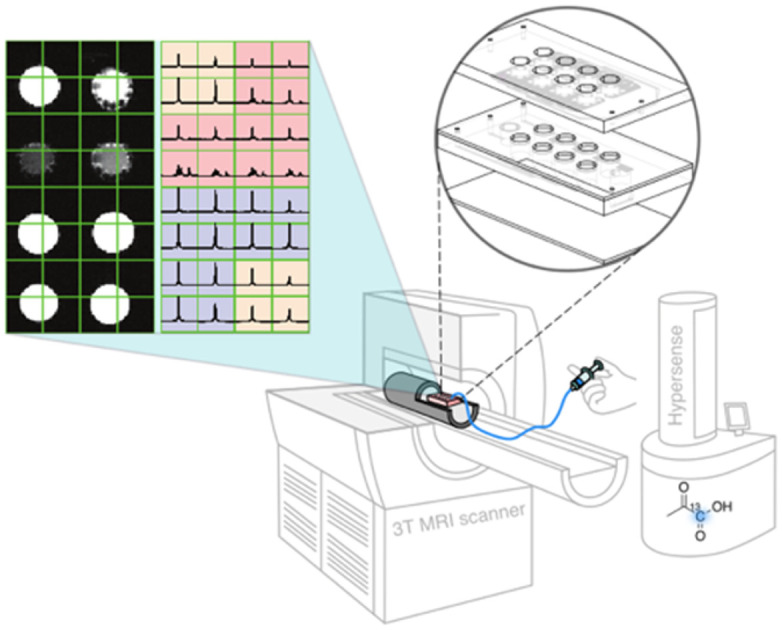

## Introduction

Dissolution dynamic nuclear polarization (dDNP) is a hyperpolarization (HP) method that enhances the sensitivity of magnetic resonance (MR) at thermal equilibrium by more than 10 000 times^1^ and enables real-time, *in situ* monitoring of chemical reactions^2^ with a time resolution of seconds and nanomolar sensitivity.^3^ There is a large variety of NMR techniques to study chemical reaction kinetics. For instance, time-resolved measurements can be made by taking a sequence of spectra when the chemical dynamics are slower than the acquisition time of the NMR free induction decay (FID) — or by time-of-flight in-flow measurements in the opposite situation when chemical dynamics are relatively fast.^4^ Metabolic reactions that typically occur on the same time scale as the relaxation time of HP ^13^C spins fall into the former category.

Unlike other HP techniques such as parahydrogen induced polarization^5,6^ or chemically induced dynamic nuclear polarization,^7^ the strength of dDNP lies in the wide variety of biocompatible hyperpolarized substrates that reveal key metabolic pathways untraceable by any other technique.^8,9^ Moreover, dDNP is the only HP-MR technique currently undergoing clinical investigation for safety and for diagnosis and post-treatment assessment.^10,11^

The dDNP process involves polarization transfer from free electrons to the sample nuclear spins, increasing the nuclear spin population in the lower energetic state, and therefore the corresponding NMR signal. To achieve this, the sample must be cooled to liquid helium temperatures (∼1.4 K) and placed in a strong magnetic field (>3 T). Microwaves are then used to excite the free electrons and transfer their polarization to the nuclear spins of interest.

Advances in dDNP-MR have improved polarization efficiency,^12,13^ chemical formulations to avoid persistent radicals in the sample^14–17^ and lifetime of spin hyperpolarization in the solid state for sample transportation.^3,18,19^ However, the application potential of dDNP-MR is still limited by the time-consuming polarization build-up process and fast relaxation of the nuclear magnetization post dissolution. For instance, polarization time of [1-^13^C]pyruvate is usually on the order of tens of minutes, while *T*_1_ relaxation is on the order of tens of seconds at room temperature.^20^

Reactants and products in chemical reactions can be detected with a time resolution of seconds and nanomolar sensitivity using dissolution dDNP-MR.^15^ A major drawback of the technique, however, is that the preparation of hyperpolarized substrates by dDNP is time-consuming and costly, which limits its ability to generate data. A minimum amount of hyperpolarized sample has to be produced as an equipment limitation, and usually not more than 3–10% of the sample is used in the experiment.^21^ The development of more efficient methods to fully utilize the polarized samples, like the one proposed here, will shorten experimental time, increase throughput, decrease its associated costs, improve control over experimental variables and reproducibility and, as a result, contribute to expand the applications of dDNP-MR.

Commercial MR instrumentation only allows single sample acquisitions or a handful of consecutive samples as the polarization decays. Repeated MR readings have been possible with a single dDNP sample by continuous filling of a volume microcoil^8^ or sequential acquisitions by removing, reloading, and introducing a new sample again in a MR imaging (MRI) scanner and a high field NMR spectrometer.^3,22–24^ However, consecutive acquisitions are not conducted under the same hyperpolarized substrate conditions (*i.e.* polarization level, substrate concentration and pH may vary between DNP dissolutions) and may be affected by *B*_0_ distortions (shim maladjustments), which affect data repeatability and make comparison between sample groups impractical. An alternative strategy to effectively shorten the polarization build-up cycle is simultaneous polarization of multiple samples. Currently in vivo dDNP-MR experiments are typically limited to four samples, which are dissolved one by one on demand, for [1-^13^C]pyruvate injections at 5–20 min intervals.^14,25^ However, despite these advances, the throughput of dDNP remains long and inefficient. A relatively large volume of hyperpolarized sample (∼4.5 mL) is produced in each polarization process, while only a few hundred microliters are usually consumed, and the remaining sample is discarded.

This project was designed to address the unmet need for technology which enables parallel dDNP-MR experiments using a single spectrometer and demonstrate that microfluidics can effectively enhance the throughput of a single dDNP shot and provide many replicates for comparing experimental parameters. An MRI-compatible microfluidic structure was produced containing eight sample wells, each independently and uniformly infused with spin-hyperpolarized solution and vital media. All 8 chambers can be probed with a single dDNP sample using the same MR acquisition, with consistent and uniform polarization conditions between sample chambers. As a demonstration of the technology, dDNP-MR spectroscopic images (dDNP-MRSI; *i.e.* MRI encoding of space with a chemical shift dimension) were acquired using commercially available RF coils in a preclinical 3 T MRI scanner, where we detected reactants and products generated from a pyruvate decarboxylation *via* hydrogen peroxide (H_2_O_2_).^26^ Three reaction replicates and five control samples were tested simultaneously in the device.

## Methods

### Fabrication of the microfluidic platform for DNP-MR applications

Molds with microfluidic channels and features were fabricated by standard photolithography methods.^27^ Silicon wafers (4′′ n-type <111>, MicroChemicals GmbH) were dehydrated using a hot plate (200 °C for 30 min) and treated with O_2_ plasma (PDC-002, Harrick Plasma) at 22.5 mL min^−1^ and 30 W for 20 min. Photoresist (SU-8 2100, KAYAKU Advanced Materials, Inc.) was spin-coated in two steps (500 rpm for 10 s with acceleration of 100 rpm s^−1^ followed by 1600 rpm for 30 s with 300 rpm s^−1^) to form a SU-8 layer of 200 μm thickness. The wafer was then soft-baked in two steps, firstly at 65 °C for 5 min, and secondly at 95 °C for 40 min. Photoresist was finally exposed to UV-light at 320 mJ cm^−2^ (17.5 mW cm^−2^ for 18.2 s) using a mask aligner machine (MJB4, Süss MicroTec) and post-baked at 65 °C for 5 min followed by 95 °C for 14 min. SU-8 structures were developed in SU-8 developer (MicroChemicals GmbH) for 16 min, rinsed with isopropyl alcohol, and dried with a N_2_ gun. The wafers were finally hard-baked at 95 °C for 30 min and silanized by vapor exposure of 1*H*,1*H*,2*H*,2*H*-perfluorooctyl-trichlorosilane (PFOTS, 97%, Merck) in a vacuum desiccator for 1 h.

PDMS layers of the microfluidic device were fabricated by soft lithography protocols using the patterned SU-8 molds. PDMS prepolymer (Sylgard 184, Ellsworth Adhesives) was prepared in a ratio of 10 : 1 (elastomer base: curing agent, w/w) and degassed in a vacuum desiccator. The prepolymer was then cast on a Petri dish containing the SU-8 mold, baked at 65 °C for 4 h, and left overnight at room temperature. PDMS layers obtained included the microfluidic channels for 1) distributing the hyperpolarized sample (upper 4 mm thick layer), 2) suctioning fluid from the chambers (intermediate 1 mm thick layer), and 3) infusing fluid into the chambers (lower 5 mm thick layer). Chambers and ports (inlets and outlets) were punched in the corresponding PDMS layers, using 6 mm and 1.25 mm biopsy punches respectively. The three layer and a glass slide (75 × 38 mm Corning) were activated using O_2_ plasma (PDC-002, Harrick Plasma) and bonded together, resulting in a device with final dimensions of 11 mm thickness, 75 mm length, and 38 mm width.

To operate the microfluidic device from outside the MRI scanner, the inlet port of the microfluidic network for injecting the hyperpolarized sample, was connected to a female Luer-to-barb fitting using 114 cm of polytetrafluoroethylene (PTFE) tubing (1/16′′ outer diameter × 1/32′′ internal diameter Darwin microfluidics). This allowed the fast administration of hyperpolarized solutions by means of a syringe. Remaining ports (*i.e.*, infusion and withdrawal ports) were connected with the same tubing and closed using tubing clamps.

### Hyperpolarization of [1-^13^C]pyruvate

A volume of 24 μL of [1-^13^C]pyruvic acid (Sigma Aldrich, Munich, Germany) containing 15 mM trityl radical OX063 (GE healthcare) and 1.5 mM Dotarem (Guerbet, Villepinte, France) were inserted into a commercial dissolution-DNP polarizer (HyperSense, Oxford Instruments Ltd.). This sample was polarized for approximately 55 min using 100 mW microwaves at 94.095 GHz in a magnetic field of 3.35 T, reaching a polarization level of around 15–20%. The hyperpolarized sample was dissolved in 4.5 mL of heated phosphate buffered saline supplemented with 1% HEPES, 0.01% EDTA, 0.1% NaCl, and 0.2% NaOH (pH 12), yielding 80 mM [1-^13^C]pyruvate and 80 μM trityl radical at pH 7.

### MRI data acquisition and hyperpolarized ^13^C-MRS

MR spectra were obtained from each individual chamber in the microfluidic platform by MR spectroscopic imaging (MRSI) techniques using a dual-tuned ^1^H–^13^C volume coil (42 mm inner diameter, Bruker) inside a horizontal 3 T MRI scanner (BioSpec 105 mm bore diameter, Bruker®). Magnetic field inhomogeneities were corrected with a shimming process applied to a water-filled device prior to the DNP-MRSI assays.

Hyperpolarized sample, 1.6 mL, was injected into the microfluidic platform, which resulted in 125 ± 5 μL delivered in each chamber over a period of 3 seconds. Hyperpolarized ^13^C MR data was acquired with a chemical shift imaging (CSI) sequence (8 × 8 matrix, field of view of 40 mm × 40 mm, area of 5 mm × 5 mm per voxel, slice thickness of 12 mm, 15° flip angle, echo time of 1.49 ms, *T*_acq_ of 51.2 ms, and RT of 66.907 ms). Each point of the data set was acquired every 4 seconds. MR *T*_2_-weighted images were acquired using 4 scans. The whole matrix of voxels (*i.e.*, 64 spectra) were acquired every 4 s with the first scan at the end of the hyperpolarized solution injection, *i.e.* at time 0. The transfer time between dissolution and injection was around 12 s.

The thermal equilibrium acquisition was performed on the microfluidic device with 100 μL solution of 8 M [^13^C]urea (Sigma Aldrich, Munich, Germany) and 10 mM Dota-Gd in two chambers located in the corners of the chip (Fig. 3A, highlighted in yellow). Those chambers were used to center the offset frequency, perform *B*_1_ calibration, shimming, and signal to noise ratio (SNR). Three chambers were filled with an aqueous solution containing 8.2 M H_2_O_2_ (Sigma Aldrich, Munich, Germany) and 37 mM NaOH (Fig. 3A, highlighted in red). The three remaining chambers were filled with 35 μL deionized water and 6.4 μL of 0.25 M NaOH solution (Fig. 3A, highlighted in blue). The MR localizer was performed with axial, sagittal, and coronal *T*_2_-weighted images using a spin echo *T*_2_ TurboRare sequence to assess signal location and accurately program the CSI voxels matrix.

### MRI data processing

Resulting CSI data were reconstructed and visualized in SIVIC.^28^

To determine the MR signal from each chamber, spectra from the voxels containing regions of the same chamber were summed. The *T*_2_ weighted image was used to select those voxels, and a total of 4 voxels were summed per each chamber.

## Results & discussion

### Microfluidic multiwell platform for parallel detection of chemical reactions by magnetic resonance

The microfluidic platform was designed using a CAD software 1) to be MR-compatible (*i.e.* made of non-magnetic materials), 2) to withstand a wide range of chemical reagents, solvents, and pHs, and 3) to be used in commercially available MRI hardware (*i.e.* to fit inside a 42 mm inner diameter volumetric RF coil and remain within the boundaries of the homogeneous *B*_0_ region of a preclinical 3 T MRI scanner) for improved usability and ease of implementation.

**Fig. 1:**
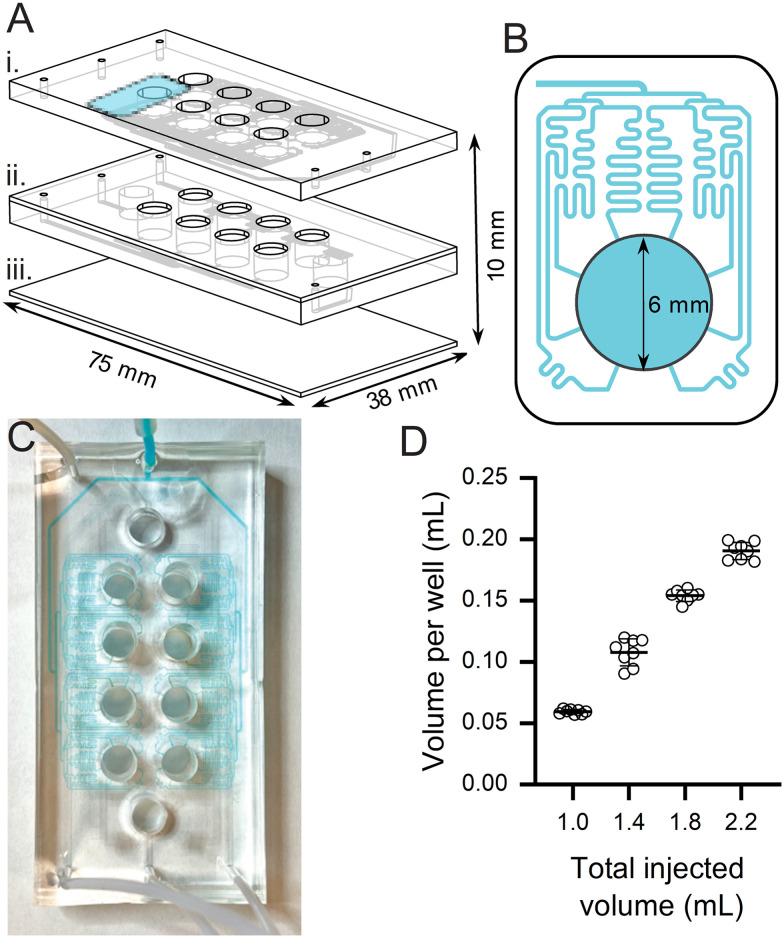
Microfluidic multiwell plate for high-throughput DNP-MRSI experiments. (A) Assembling layers of the multiwell plate: i) 4 mm thick PDMS layer for distribution of the hyperpolarized substrate, ii) 5 and 1 mm thick PDMS layers with the detection chambers, and iii) 75 mm × 38 mm glass slide on the bottom of the PDMS chambers as a support. (B) Network of microfluidic channels that injects the polarized sample into one of the chambers. This design is replicated along all the chambers. (C) Photo of the microfluidic device. Microfluidic channels that distribute the polarized sample are filled with blue dye for easy identification. (D) Water volume inside each chamber after injecting 1.0, 1.4, 1.8, and 2.2 mL water into the device (each circle represents a chamber of the microfluidic plate).

The platform was engineered using microfabrication (photolithography and soft lithography) processes and consisted of a stack of three polydimethylsiloxane (PDMS) layers on a glass substrate (Fig. 1A).^29^ The PDMS layers were developed by replica molding using a photoresist-patterned mold and were dedicated to the microfluidic parts: the sample-containing 4 × 2 array of 6 mm-diameter chambers (of 15 mm center to center spacing, 10 mm height, ∼280 μL total volume) and the microfluidic channels to distribute the hyperpolarized solution among the chambers. A network of sinusoidal channels (Fig. 1B), acting as fluidic resistances, in the upper PDMS layer split the sample throughout the device and made it possible to deliver the hyperpolarized solution rapidly and simultaneously to all the chambers. Eight distributed microfluidic channels leading to each chamber were designed to deliver the hyperpolarized sample from the top of the chamber to the testing sample, facilitating a rapid delivery of the fluid under 4 s (video in ESI† material).

Two further PDMS layers with microfluidic channels were integrated between the above layers to continuously infuse and withdraw solutions into and from the chambers. These channels were grouped in 2 independent sets of 4 chambers that shared inlet and outlet ports (enabling 2 different conditions and 4 replicates when these channels are open). These allow for a myriad of experimental setups in future applications. For example, they can be used for continuous renewal of the solution in the chambers while the device is located inside the MRI scanner. In this work, all experiments were performed with the withdrawal circuits closed in the device shown in Fig. 1C.

To confirm uniform distribution of samples across the chambers, we injected different volumes of water throughout the inlet port and collected and weighed the liquid received in each chamber. For total injected volumes of 1 mL, 1.4 mL, 1.8 mL, and 2.2 mL, the volumes per chamber were 59 ± 2 μL, 108 ± 11 μL, 154 ± 5 μL, and 191 ± 7 μL, respectively (Fig. 1D). Applying linear regression, the data fit (*R*-squared of 0.96) the following equation:1
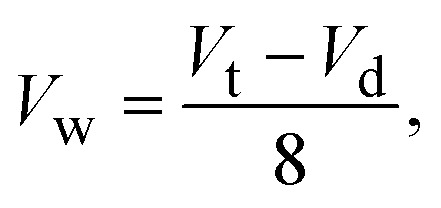
where *V*_w_ is the volume per well, *V*_t_ is the total injected volume, and *V*_d_ is the dead volume (576 ± 69 μL) mainly due to the long fluidic tubing required for injecting from outside of the MRI scanner. This confirmed that the injected solutions arrived at each well at the exact same time and with equal volume through the fluidic channels.

**Fig. 2:**
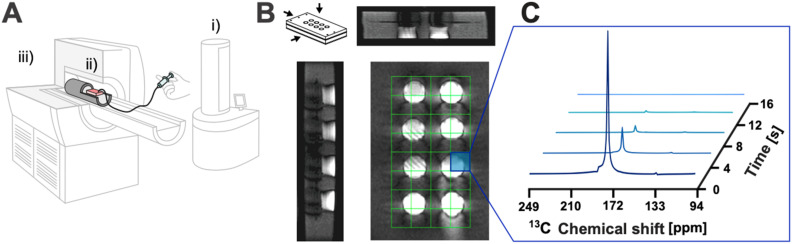
High-throughput DNP-MRSI assay. (A) Schematic illustration of the experimental setup consisted of i) polarizer equipment, ii) microfluidic multiwell plate, and iii) magnetic resonance imaging scanner. (B) *T*_2_-weighted localizer images with axial, sagittal and coronal perspectives. (C) Stacked dynamic spectra of [1-^13^C]pyruvic acid of the well highlighted in blue along the experiment. Note that time 0 represents when the MRS data acquisition was initiated. The transfer time between dissolution and injection was 12 s on average, and MRS data acquisition was initiated 25 s after the sample came out from the polarizer. The longitudinal experiment was designed with a CSI sequence as 8 × 8 voxels matrix, with a FOV of 40 × 40 cm^2^ and a slice thickness of 12 mm, 15° flip angle, echo time = 1.49 ms. *T*_acq_ = 51.2 ms and RT = 66.907 ms. Each point of the data set was acquired every 4 seconds.

In order to increase the throughput of HP experiments by a factor of 8 using our microfluidic device, we exploited the potential of MR spectroscopic imaging (MRSI), by which spatial and spectral information can be obtained simultaneously (Fig. 2A). To test the device for HP-MRSI, a solution of 80 mM hyperpolarized [1-^13^C]pyruvate was injected through the fluidic channels. The matrix (8 × 8) and voxel size (5 mm × 5 mm) was selected to cover all the chambers while maintaining shimming performance (Fig. 2B). Although the voxel size was smaller than the chamber size, we found that multiple voxels per chamber simplified the alignment of the device inside the RF coil. Since no voxel included more than one chamber, analysis was performed by adding the signals from voxels encompassing the same chamber.

Fig. 2C shows a representative spectrum from one voxel that contains part of a chamber. The apparent relaxation time constant of [1-^13^C]pyruvate was 3.7 ± 0.7 s, as calculated from the exponential decay of the C1 pyruvate resonance at 176 ppm chemical shift. Although the *T*_1_ of [1-^13^C]pyruvate after dDNP is expected to be around 60 s,^30^ the repeated RF pulses used to obtain the spectroscopic images (*i.e.*, 64 pulses per each scan) caused a much faster ^13^C polarization loss. The spectral linewidth of the ^13^C1 pyruvate peak was 1.8 ppm (53 Hz) at full width at half maximum height.

The axial, sagittal, and coronal proton *T*_2_ weighted MR images acquired after the HP ^13^C-MRSI experiment confirmed that the HP solution had been delivered to all the chambers as expected (Fig. 2B).

Contrary to previous studies that increased the throughput either by sequential measurements with the same dissolution sample^3,8^ or by parallel polarization of 4 samples,^14,25^ our MRSI approach improves repeatability as all tested samples are detected under the same hyperpolarized substrate and shim conditions (FWHM = 53 ± 5 Hz).

### Simultaneous detection of eight chemical reaction processes: decarboxylation of hyperpolarized [1-^13^C]pyruvate and controls

[1-^13^C]pyruvate, a key metabolite in glycolysis^31^ and a cellular antioxidant agent capable of neutralizing peroxides by decarboxylation,^32,33^ is extensively used in dDNP-MR experiments due to its long *T*_1_ relaxation time constant (in excess of 1 minute)^30^ and high polarization (up to 36% with a SpinLab™ polarizer®),^34^ which provide MR signals of both pyruvate and its products.

**Fig. 3:**
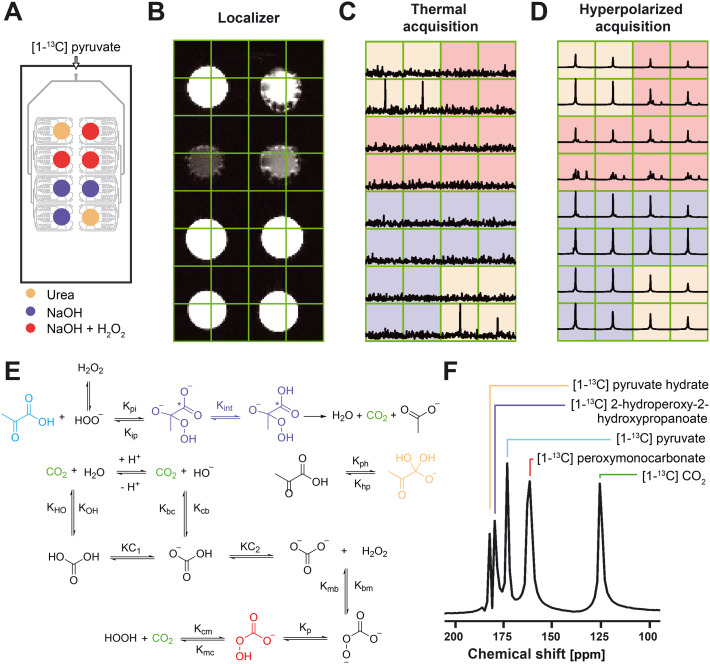
(A) Schematic image of the microfluidic device specifying the sample placed in each well. (B) Localizer with a coronal *T*_2_-weighted image using a spin echo *T*_2_ TurboRARE sequence with the corresponding voxels matrix overlapping. (C) Spectroscopic acquisition using a CSI pulse sequence to the microfluidic device at thermal equilibrium. (D) First spectroscopic acquisition point using a CSI pulse sequence to the microfluidic device after the hyperpolarized [1-^13^C]pyruvate injection. (E) Scheme of the oxidation reaction between pyruvic acid and H_2_O_2_, highlighting all products and intermediates displayed in the spectrum. (F) Representative spectrum of the decarboxylation of [1-^13^C]pyruvate reacted with H_2_O_2_, showing all the products and intermediate states of the reaction.

As a proof of concept for detecting fast reactions using our parallelized microfluidic approach, we traced the ^13^C resonance shifts of the biologically relevant chemical reaction between [1-^13^C]pyruvate and H_2_O_2_ by acquiring spatially-localized ^13^C-MR spectra of the whole device every 4 seconds. Using the entire chamber capacity of the platform, two chambers of the platform were filled with a [^13^C]urea solution for chamber arrangement verification, three with a H_2_O_2_ solution to initiate the chemical reaction, and three with a sodium hydroxide solution as a negative control of the reaction (Fig. 3A).

**Fig. 4:**
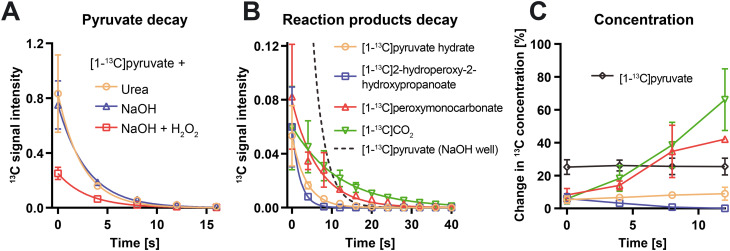
(A) Plot showing the temporal evolution of the peak signal intensity, normalized by the signal intensity and volume of [1-^13^C]pyruvate, in wells containing [^13^C]urea (orange circle), wells containing H_2_O_2_ and sodium hydroxide solution (red square) and wells containing water and sodium hydroxide solution (blue triangle). (B) Plot showing the temporal evolution of the normalized peak signal intensity of [1-^13^C]pyruvate hydrate (orange circle), [1-^13^C]2-hydroperoxy-2-hydroxypropanoate (blue square), [1-^13^C]peroxymonocarbonate (red triangle), and ^13^CO_2_ (inverted green triangle) over time in wells containing H_2_O_2_ and sodium hydroxide solution. Normalized peak signal intensity of [1-^13^C]pyruvate (dashed line) in well containing only sodium hydroxide is included in dashed line as a reference. The signal intensity displayed in each of the four voxels corresponding to a well were summed up and each well corresponds to a replicate. (C) Temporal evolution of the change in concentration of the reactants and products with respect to their initial concentration in the wells containing H_2_O_2_ and sodium hydroxide solution, corrected by the hyperpolarized signal *T*_1_ decay. The legend is the same as in (B).

The placement of the microfluidic device and acquisition matrix was validated before to the HP experiment by a proton *T*_2_-weighted image (Fig. 3B) and again after by detecting the thermal signal of the [^13^C]urea solution from two opposite corners of the device (Fig. 3C). After injection of the hyperpolarized pyruvate solution, the spectra of the chamber regions without H_2_O_2_ displayed only the pyruvate peak since no reaction had occurred (Fig. 3D). In chambers where the reaction took place, the bubbles generated by CO_2_ as a side of the reaction were easily visible, and spectral acquisitions displayed the characteristic multi-peak spectrum of pyruvate decarboxylation^18^ (Fig. 3E and F), where the following peaks were identified: [1-^13^C]pyruvate (176 ppm), [1-^13^C]pyruvate hydrate (184 ppm), [1-^13^C]2-hydroperoxy-2-hydroxypropanoate (181 ppm), [1-^13^C]peroxymonocarbonate (161 ppm), and ^13^CO_2_ (125 ppm). Parallel MRSI in the preclinical MRI scanner achieves high throughput at the expense of some spectral resolution, in comparison to say, a dedicated NMR spectrometer. The trade-off in resolution is, however, more than acceptable for hyperpolarized ^13^C MRSI where the resonances of metabolic products are dispersed over a wide chemical shift range. Additionally, the temporal evolution of the [1-^13^C]pyruvate signal was compared between different conditions by averaging the signal intensities from replicated chambers (Fig. 4). Dispersion among the three chambers per condition for the pyruvate peak were 23% and 18% at the first acquisition for chambers with and without the chemical reaction, respectively. At the time of the first acquisition (*i.e.*, 25 s from dissolution), the pyruvate peak intensity from chambers with H_2_O_2_ was 2.5–3 lower than the signal from chambers containing non-oxidant solutions because of pyruvate's rapid consumption during the reaction (Fig. 4A). The pyruvate signal decay was dominated by the RF pulse polarization consumption, with an apparent decay time constant of 3.03 s in chambers with H_2_O_2_ and 2.96 s in chambers without reactant.

The observation that the relaxation time constant of [1-^13^C]pyruvate was similar (∼3 s) among conditions (*i.e.*, chambers with and without H_2_O_2_) indicates that the resulting decay was exclusively due to the signal exponential decay by *T*_1_ and signal depletion by RF pulsing 64 times/acquisition. This also suggests that practically all the pyruvate had already been converted into the intermediate at the time of the first acquisition. In the chambers with H_2_O_2_, the signals from reaction subproducts decayed as a result of both the generation of the reactant and the *T*_1_ relaxation time constant of the hyperpolarized carbon nuclei (Fig. 4B). While over-crowded peaks in the downfield region (pyruvate, its hydrate and 2-hydroperoxy-2-hydroxypropanoate) could be detected above the noise level (*i.e.*, SNR > 5) for up to 33 s after the injection of hyperpolarized pyruvate, the upfield peaks of the two final products (^13^CO_2_ and [1-^13^C]peroxymonocarbonate) were detectable for over a minute. The longer observation window for the products is probably attributed to the oxidation reaction, as the ongoing production of the final molecules counteracts the signal decay by *T*_1_. This could account for the slower decay rate of the two generated products (*i.e.*, [1-^13^C]peroxymonocarbonate and ^13^CO_2_) respect to the consumed reactant and intermediate compound (*i.e.*, [1-^13^C]pyruvate and [1-^13^C]2-hydroperoxy-2-hydroxypropanoate). Apparent decay rates were 0.28 ± 0.03 s^−1^ for [1-^13^C]pyruvate hydrate, 0.50 ± 0.05 s^−1^ for [1-^13^C]2-hydroperoxy-2-hydroxypropanoate, 0.15 ± 0.01 s^−1^ for [1-^13^C]peroxymonocarbonate, and 0.09 ± 0.01 s^−1^ for ^13^CO_2_. The time-normalized mass sensitivity is defined as2
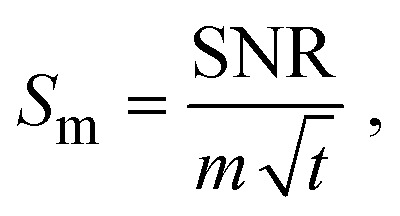
where *m* is the amount of substance in mol and *t* is the measurement time in seconds.^35^ In systems with small volumes and integrated solenoidal microcoils, which provide strong signals, *S*_m_ is in the range of thousands (∼1000–3000) μmol^−1^ s^−1/2^.^36,37^ In our experiment, the SNR and *S*_m_ of one single scan (*t* = 4 s) for pyruvate detection were 4229 and 352 μmol^−1^ s^−1/2^, respectively. This SNR is 56 times higher than the SNR obtained from a thermal polarization measurement with 65 h of averaging time with the same experimental setup and acquisition parameters.

The concentrations of all the species in the reaction were quantified considering a uniform ^13^C polarization decay (*i.e.*, decay of the pyruvate signal in the well without H_2_O_2_) among all the species. Although the *T*_1_ values of the products are likely to be slightly different, this simplification enables to determine, approximately, their concentration when *T*_1_ is unknown. Within the time period where the SNR of the pyruvate was high (first 12s), we determined a plateau of the [1-^13^C]pyruvate concentration, a decrease of the intermediate product ([1-^13^C]2-hydroperoxy-2-hydroxypropanoate), and an increase of the final products ([1-^13^C]peroxymonocarbonate, and ^13^CO_2_).

To monitor fast reactions by HP-MR, the HP solution must mix with the reactant under study within seconds of reaching the science chambers. Our microfluidic device addressed this constraint by delivering the HP solution to each chamber in a cascade mode through eight channels (Fig. 1). For experiments where samples may need a convective flow for a proper mixing, we speculate that alternate infusion and withdrawal of the testing sample in the chambers using the additional channels included in our device would further enhance the mixing with the polarized sample.

Hyperpolarization-enhanced MR techniques are rapidly evolving and are expected to transform current analytical tools. Increasing their throughput will help overcome the barrier that prevents these techniques from being more accessible. This work showed that combining dDNP-MRSI and microfluidics can increase the capacity of identify products from chemical reactions and demonstrated that multiple samples can be simultaneously and non-invasively interrogated. Reaction kinetics and *in situ* metabolomic studies are potential applications of our platform beyond the proof-of-principle in this work.

## Author contributions

J. Y. designed the microfluidic device and wrote the manuscript. M. A. designed and performed the experiments, analyzed the data, and wrote the manuscript. M. A. O., A. P., and G. M. designed, fabricated, and tested the microfluidic device. A. H. G. performed experiments and analyzed the data. Y. K., R. S., J. K., and D. B. V. conducted the MRI experiments. I. M. R. designed the experiments, raised funding, supervised the work, wrote, and reviewed the manuscript.

## Conflicts of interest

I. M. R. and M. A. O. are co-founders of Vitala Technologies S. L. IBEC has filed a patent application related to this work. M. A., J. Y., M. A. O., A. H. G. and I. M. R. are co-inventors of this patent application. The authors declare that they have no other competing interests.

## Supplementary Material

LC-023-D3LC00474K-s001

LC-023-D3LC00474K-s002

## References

[cit1] Ardenkjær-Larsen J. H., Fridlund B., Gram A. (2003). *et al.* Increase in signal-to-noise ratio of >10,000 times in liquid-state NMR. Proc. Natl. Acad. Sci. U. S. A..

[cit2] Zeng H., Lee Y., Hilty C. (2010). Quantitative rate determination by dynamic nuclear polarization enhanced NMR of a diels-alder reaction. Anal. Chem..

[cit3] Lees H., Millan M., Ahamed F. (2021). *et al.* Multi-sample measurement of hyperpolarized pyruvate-to-lactate flux in melanoma cells. NMR Biomed..

[cit4] Patra B., Sharma M., Hale W., Utz M. (2021). Time-resolved non-invasive metabolomic monitoring of a single cancer spheroid by microfluidic NMR. Sci. Rep..

[cit5] Buntkowsky G., Theiss F., Lins J. (2022). *et al.* Recent advances in the application of parahydrogen in catalysis and biochemistry. RSC Adv..

[cit6] Eills J., Cavallari E., Carrera C., Budker D., Aime S., Reineri F. (2019). Real-Time Nuclear Magnetic Resonance Detection of Fumarase Activity Using Parahydrogen-Hyperpolarized [1-^13^C]Fumarate. J. Am. Chem. Soc..

[cit7] Sheberstov K. F., Chuchkova L., Hu Y. (2021). *et al.* Photochemically Induced Dynamic Nuclear Polarization of Heteronuclear Singlet Order. J. Phys. Chem. Lett..

[cit8] Jeong S., Eskandari R., Park S. M. (2017). *et al.* Real-time quantitative analysis of metabolic flux in live cells using a hyperpolarized micromagnetic resonance spectrometer. Sci. Adv..

[cit9] Keshari K. R., Wilson D. M. (2014). Chemistry and biochemistry of ^13^C hyperpolarized magnetic resonance using dynamic nuclear polarization. Chem. Soc. Rev..

[cit10] Home – ClinicalTrials.gov, Accessed January 9, 2023, https://clinicaltrials.gov/

[cit11] Vaeggemose M., Schulte R. F., Laustsen C. (2021). Comprehensive Literature Review of Hyperpolarized Carbon-13 MRI: The Road to Clinical Application. Metabolites.

[cit12] Chaudhari S. R., Wisser D., Pinon A. C. (2017). *et al.* Dynamic Nuclear Polarization Efficiency Increased by Very Fast Magic Angle Spinning. J. Am. Chem. Soc..

[cit13] Elliott S. J., Cala O., Stern Q. (2021). *et al.* Boosting dissolution-dynamic nuclear polarization by multiple-step dipolar order mediated 1H→13C cross-polarization. J. Magn. Reson. Open.

[cit14] Krajewski M., Wespi P., Busch J. (2017). *et al.* A multisample dissolution dynamic nuclear polarization system for serial injections in small animals. Magn. Reson. Med..

[cit15] Su Y., Andreas L., Griffin R. G. (2015). Magic Angle Spinning NMR of Proteins: High-Frequency Dynamic Nuclear Polarization and 1H Detection. Annu. Rev. Biochem..

[cit16] Marco-Rius I., Cheng T., Gaunt A. P. (2018). *et al.* Photogenerated Radical in Phenylglyoxylic Acid for in Vivo Hyperpolarized ^13^C MR with Photosensitive Metabolic Substrates. J. Am. Chem. Soc..

[cit17] Gaunt A. P., Lewis J. S., Hesse F. (2022). *et al.* Labile Photo-Induced Free Radical in α-Ketoglutaric Acid: a Universal Endogenous Polarizing Agent for In Vivo Hyperpolarized ^13^C Magnetic Resonance. Angew. Chem..

[cit18] Capozzi A. (2022). Design and performance of a small bath cryostat with NMR capability for transport of hyperpolarized samples. Sci. Rep..

[cit19] El Daraï T., Cousin S. F., Stern Q. (2021). *et al.* Porous functionalized polymers enable generating and transporting hyperpolarized mixtures of metabolites. Nat. Commun..

[cit20] Marco-Rius I., Tayler M. C. D., Kettunen M. I. (2013). *et al.* Hyperpolarized singlet lifetimes of pyruvate in human blood and in the mouse. NMR Biomed..

[cit21] Ros S., Wright A. J., Bruna A., Caldas C., Brindle K. M. (2021). Metabolic imaging with hyperpolarized [1-^13^C]pyruvate in patient-derived preclinical mouse models of breast cancer. STAR Protoc..

[cit22] Eills J., Hale W., Sharma M., Rossetto M., Levitt M. H., Utz M. (2019). High-Resolution Nuclear Magnetic Resonance Spectroscopy with Picomole Sensitivity by Hyperpolarization on a Chip. J. Am. Chem. Soc..

[cit23] Lee J., Ramirez M. S., Walker C. M. (2015). *et al.* High-throughput hyperpolarized ^13^C metabolic investigations using a multi-channel acquisition system. J. Magn. Reson..

[cit24] Kim Y., Liu M., Hilty C. (2018). Determination of binding affinities using hyperpolarized NMR with simultaneous 4-channel detection. J. Magn. Reson..

[cit25] Hu S., Larson P. E. Z., VanCriekinge M. (2013). *et al.* Rapid sequential injections of hyperpolarized [1-^13^C]pyruvate in vivo using a sub-kelvin, multi-sample DNP polarizer. Magn. Reson. Imaging.

[cit26] Drachman N., Kadlecek S., Duncan I., Rizi R. (2017). Quantifying reaction kinetics of the non-enzymatic decarboxylation of pyruvate and production of peroxymonocarbonate with hyperpolarized ^13^C-NMR. Phys. Chem. Chem. Phys..

[cit27] Qin D., Xia Y., Whitesides G. M. (2010). Soft lithography for micro- and nanoscale patterning. Nat. Protoc..

[cit28] Crane J. C., Olson M. P., Nelson S. J. (2013). SIVIC: Open-Source, Standards-Based Software for DICOM MR Spectroscopy Workflows. Int. J. Biomed. Imaging.

[cit29] Friend J., Yeo L. (2010). Fabrication of microfluidic devices using polydimethylsiloxane. Biomicrofluidics.

[cit30] Chattergoon N., Martínez-Santiesteban F., Handler W. B., Ardenkjær-Larsen J. H., Scholl T. J. (2013). Field dependence of T1 for hyperpolarized [1-^13^C]pyruvate. Contrast Media Mol. Imaging.

[cit31] Guarino V. A., Oldham W. M., Loscalzo J., Zhang Y. Y. (2019). Reaction rate of pyruvate and hydrogen peroxide: assessing antioxidant capacity of pyruvate under biological conditions. Sci. Rep..

[cit32] Mallet R. T., Squires J. E., Bhatia S., Sun J. (2002). Pyruvate restores contractile function and antioxidant defenses of hydrogen peroxide-challenged myocardium. J. Mol. Cell. Cardiol..

[cit33] Desagher S., Glowinski J., Prémont J. (1997). Pyruvate Protects Neurons against Hydrogen Peroxide-Induced Toxicity. J. Neurosci..

[cit34] Ardenkjaer-Larsen J. H., Leach A. M., Clarke N., Urbahn J., Anderson D., Skloss T. W. (2011). Dynamic nuclear polarization polarizer for sterile use intent. NMR Biomed..

[cit35] Lacey M. E., Subramanian R., Olson D. L., Webb A. G., Sweedler J. V. (1999). High-Resolution NMR Spectroscopy of Sample Volumes from 1 nL to 10 μL. Chem. Rev..

[cit36] Olson D. L., Peck T. L., Webb A. G., Magin R. L., Sweedler J. V. (1995). High-Resolution Microcoil 1H-NMR for Mass-Limited, Nanoliter-Volume Samples. Science.

[cit37] Mompeán M., Sánchez-Donoso R. M., De La Hoz A., Saggiomo V., Velders A. H., Gomez M. V. (2018). Pushing nuclear magnetic resonance sensitivity limits with microfluidics and photo-chemically induced dynamic nuclear polarization. Nat. Commun..

